# Locomotor characteristics of intense accelerations according to the playing position in top Spanish football teams during competition

**DOI:** 10.5114/biolsport.2025.151652

**Published:** 2025-06-24

**Authors:** Joaquín González-Rodenas, Víctor Moreno-Pérez, Adrián Castaño-Zambudio, Roberto López-Del Campo, Fabio Nevado, Juan Del Coso

**Affiliations:** 1 Sport Sciences Research Centre, Rey Juan Carlos University, Fuenlabrada, Spain; 2 Sports Research Center, Miguel Hernandez University of Elche, Alicante, Spain; 3 Department of competitions and Mediacoach, LaLiga, Madrid, Spain

**Keywords:** Physical demands, Soccer, Activity profile, High-intensity actions, Positional roles

## Abstract

This study aimed to characterise the locomotor characteristics of intense relative accelerations (> 50% of maximal acceleration-speed profile) according to playing positions in top-ranked Spanish football teams. A total of 271,535 accelerations performed by 102 football players of the top four teams during the 2023–2024 LaLiga season were registered by a multiple-camera computerised tracking system (TRACAB; ChyronHego, USA). A generalized linear mixed model compared the acceleration characteristics (number, distance, duration, intensity and speed) across playing positions (central defender: CD, full back: FB, central midfielder: CM, attacking midfielder: AM, winger: W and forward: FW) considering the effect of contextual variables. CD exhibited less intense accelerations per minute than the rest of the playing positions (p < 0.05). W exhibited the greatest distance per acceleration (estimated mean (EM) = 9.08 m), longest duration (EM = 2.18 s), highest peak speed (EM = 19.5 km · h^−1^), and highest speed at maximum acceleration (EM = 12.8 km · h^−1^) (p < 0.05). FW exhibited the highest peak acceleration per action (EM = 3.14 m · s^−2^) and average acceleration per action (EM = 1.76 m · s^−2^) (p < 0.05). AM and CM registered higher initial speed (p < 0.05) and exhibited the lowest values for peak acceleration per action (p < 0.05), compared to the other playing positions. In contrast, CD and CM showed the shortest distances and slowest speeds (p < 0.05). In conclusion, W and FW exhibited higher acceleration intensity than the rest of the playing positions, while CD and CM obtained the lowest values for distance, duration, and speed.

## INTRODUCTION

Football (soccer) is a complex team sport characterised by high physical demands that require players to perform a wide range of high-intensity actions throughout the match [1, 2]. Notably, top competitions such as the Spanish LaLiga and the English Premier League have exhibited a notable increase in physical intensity in recent years, showing a greater frequency of high-intensity efforts and greater distance covered at high intensity during matches [3, 4].

In this context, a growing body of research over the past decade has analysed the locomotor demands of elite football, shedding light on the specific running demands based on playing positions [1, 2], level of the competition [5, 6], as well as multiple contextual variables [7–9]. In many of these studies, key metrics such as total distance, high-intensity running distance and sprint distance have been used to characterise the match physical demands [10], enabling coaches to achieve optimal training targets [11].

However, it is important to note that many intense actions in football do not involve reaching high speeds, such as tackling, jumping or accelerating [12]. These actions typically occur over short distances and at low speeds and are often not classified as high-intensity in studies on the physical demands of elite football, which rely on distance covered at various speed thresholds. Despite the limited distance and speed of accelerations, these actions impose significant locomotor demands [13]. In fact, the quantification of acceleration activity may be more useful than high-intensity running for identifying fatigue during matches [14] and congested match periods [15]. Consequently, scientific analyses based solely on high-intensity running distance may limit the comprehensive understanding of match load in football [16].

Previous studies in professional football have primarily focused on the frequency and distance of accelerations during matches, with special focus on high-intensity accelerations (> 3 m · s^−2^). For instance, the study of Djaoui et al. [17] observed that professional Swiss players covered around 50 metres per minute while accelerating during competition, with 10% of those accelerations classified as high accelerations. Similarly, Morgans et al. [18] found that players from French Ligue 1 performed approximately 0.7 high accelerations per minute while English Premier League players performed on average 0.9 high accelerations per minute. In terms of playing positions, players in lateral positions such as wingers (W) and full backs (FB) accelerated more often compared to players in central positions, while central defenders (CD) seem have the lowest frequency of accelerations [19].

In this regard, the characterization of the locomotor profile of the intense accelerations performed during competition is key to completely understanding the physical challenges of football. It is important to note that the maximum acceleration rate occurs during the initial phases of sprinting, when players exhibit the greatest changes in velocity [20–22]. This means that acceleration decreases with increasing initial running speed, which is habitually ignored in studies that have quantified accelerations in professional football [23]. The assessment of the characteristics of the accelerations considering the initial speed, distance, duration and peak speed reached may be key for fitness coaches to monitor competition physical load in elite football [24].

However, scientific evidence on the specific locomotion characteristics of accelerations during competition remains limited. In this context, studies such as that of Sonderegger et al. [24] have emphasised the need for a deeper understanding of accelerations to establish more precise thresholds for monitoring training load in football. These authors highlighted the importance of considering the running speed prior to the acceleration, and individual maximal accelerative capacity instead of absolute acceleration thresholds in m · s^−2^, for accurately evaluating the intensity of short locomotor actions. The use of this new methodological approach is key for gaining detailed insight into accelerations and designing effective training scenarios [25]. Recent technological advancements, particularly the development of video-tracking systems, now allow for accurate and reliable measurements of player movements. In this manner, an exclusive analysis of accelerations may provide a more precise assessment of these high-intensity neuromuscular actions.

Therefore, this study aimed to evaluate the locomotor characteristics of intense relative accelerations (> 50% of maximal acceleration-speed profile) in terms of distance, magnitude and speed, according to playing positions in top-ranked Spanish football players.

It is hypothesised that the locomotor acceleration profiles will significantly vary based on playing position. Specifically, positions such as FB, W and FW are expected to exhibit greater distance, intensity and speed in each acceleration, starting from lower speeds, while midfielders would have lower intensity and speed but higher initial speed. Finally, CD is the position with lower expected values in accelerations. Additionally, considering individualised acceleration-speed profiles is anticipated to provide a more accurate representation of the physical demands of accelerations in elite football.

## MATERIALS AND METHODS

### Participants

This study examined a total of 271,535 intense relative accelerations performed by 102 professional football players during 152 matches during the 2023–2024 LaLiga season.

These data corresponded to all players that competed in any match of the season in the top four teams in the final ranking. This sample represents the most successful teams of the season, which qualified for the UEFA Champions League, at the top European football level. Each team completed 38 matches per season, 19 at home and 19 away. The teams had the possibility of employing 5 substitutions. All matches were played on a grass surface. To be included in the analysis, a player had to participate in at least one match for a minimum of 20 minutes, ensuring meaningful involvement in match dynamics. This threshold accounts for the tendency of substitutes to exhibit higher physical metrics than starters [26–28] due to the shorter playing time and greater freshness, which could distort overall performance averages. In alignment with previous research [29], this duration was set as the minimum requirement for inclusion in the study. Goalkeepers were excluded from the analysis due to the technical, tactical and physical differences of this playing position in comparison to the field players [30].

Players were classified into six playing positions: central defenders (CD; n = 22; match observations = 50,076), full backs (FB; n = 16; match observations = 43,636), central midfielders (CM; n = 19; match observations = 61,017), attacking midfielders (AM; n = 14; match observations = 48,882), wingers (W; n = 18; match observations = 43,653), and forwards (FW; n = 13; match observations = 24,271). Some of these players were used in different positions across the matches and the categorization was updated to the actual playing position at the beginning of the match.

**FIG. 1:**
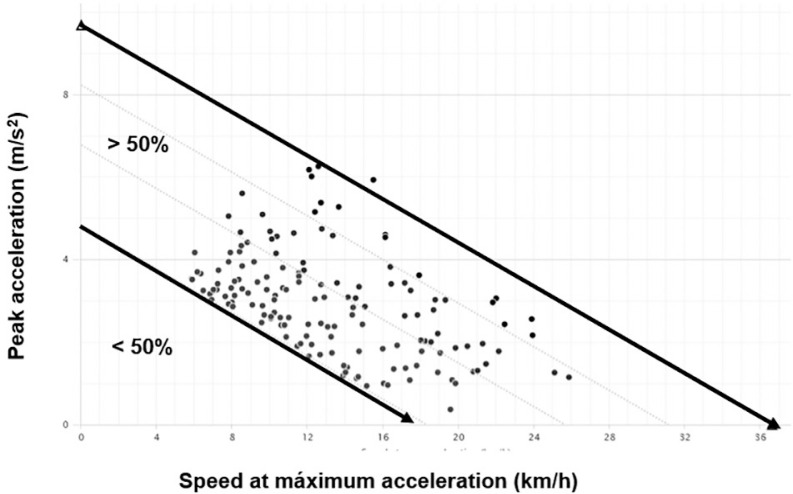
Accelerations above the 50% threshold in the accelerationspeed profile.

Specifically, the evaluated accelerations are those that exceed the 50% threshold of the acceleration-speed profile during each match (Figure 1). This inclusion criterion ensures that only accelerations with moderate intensity are considered for analysis [23], allowing for a focused examination of the upper mechanical demands of acceleration relative to the maximum speed achieved. The maximum acceleration-speed profile in football match play represents the inverse relationship between peak acceleration (Y-axis, m · s^−2^) and velocity at the point of maximum acceleration (X-axis, km · h^−1^). As velocity increases, the capacity to generate high acceleration decreases, reflecting the biomechanical trade-off between force production and movement speed (Figure 1). Specifically, only accelerations that exceeded 50% of the player’s individual maximum acceleration-speed capacity were considered for analysis. This threshold was established to ensure that the data captured represented moderately intense efforts [23, 31], which are more indicative of the physical demands associated with performance in competitive match situations. This approach provides a more precise understanding of acceleration patterns in high-intensity contexts.

In adherence to the ethical guidelines outlined by LaLiga, the present investigation does not contain any information that could lead to the identification of individual football players. Furthermore, all procedures and protocols employed in this study were conducted in accordance with the principles set forth in the Declaration of Helsinki, and the Institutional Review Board of the Rey Juan Carlos University approved this study.

### Procedure

The current investigation represents a non-experimental, descriptive, and comparative design to characterise the acceleration profile of top-ranked professional Spanish football players. The analysed matches were video captured by the TRACAB’s Gen5 optical video tracking system (Chyron, Lincoln, NE, USA), and data on accelerations and decelerations were extracted using the Mediacoach match statistics software (LaLiga, Madrid, Spain).

Briefly, TRACAB’s Gen5 consists of 16 cameras (1920 × 1080 pixels) placed at different positions in the stadium to provide a stitched panoramic image. This multicamera tracking system has been designed to identify players’ positions on the pitch and track them based on unique visual signatures for each player. TRACAB determines the X, Y (field position), and Z (height) coordinates 25 times per second and calculates players’ speed of movement as the rate of change in a player’s position over time. Curved paths are accounted for, as the system captures granular position data for non-linear motion [32].

Additionally, the data collected by TRACAB’s Gen5 in each match is further processed in Mediacoach, which includes corrections for instances where players’ tracking data may overlap [33]. In this sense, there is an operator for each match that visually corrects the situations in which the positioning coordinates are erroneous. This correction is common in corner kicks and fouls but rarely occurs during high-speed actions resulting in accelerations or decelerations. Therefore, manual corrections had minimal relevance to the objectives of this investigation. Considering this correction, real-time positional data of TRACAB’s Gen5 are used by Mediacoach to derive acceleration as the rate of change of speed over time [32]. The accuracy of this coupled TRACAB-Mediacoach system is crucial for reliably assessing physical demands, as both rapid increases in speed (accelerations) and decreases in speed (decelerations) can be detected.

The validity of TRACAB’s Gen5 for assessing movement demands during football match play has been established through high agreement with data obtained from a reference camera system (i.e., the VICON motion capture system). This system registered root mean square errors (RMSE) between 0.07 and 0.11 m (for distance) and between 0.00 and 0.02 m · s^−2^ (for acceleration) with respect to the reference criterion during football-specific actions such as small-sided games and linear and curved sprints and decelerations [32]. Additionally, the coupled TRACAB-Mediacoach system has been validated against data obtained with GPS-accelerometer devices during official football matches with coefficients of variation (CV) between 1.8 and 14.9% for total running distance and distances covered at different speeds thresholds (total distance: 1.8%; maximum speed: 4.0%; intense running: 10.4%; sprinting at high intensity: 14.9%) [34]. Also, it has been validated for total accelerations and decelerations with mean absolute percentage differences of 5.0 ± 14.9% for total accelerations and 16.6 ± 9.5% for total decelerations between the two technologies, demonstrating that TRACAB’s Gen5 is a reliable system for measuring accelerations and decelerations in professional football [35].

The acceleration demands were expressed in metres per second squared (m · s^−2^), as it allows for precise quantification of fast movements and directional changes, aligned with previous research on mechanical demands in football [23, 36]. In contrast, speed was reported in kilometres per hour (km · h^−1^), the unit widely used in the analysis of running demands in football [10].

**FIG. 2:**
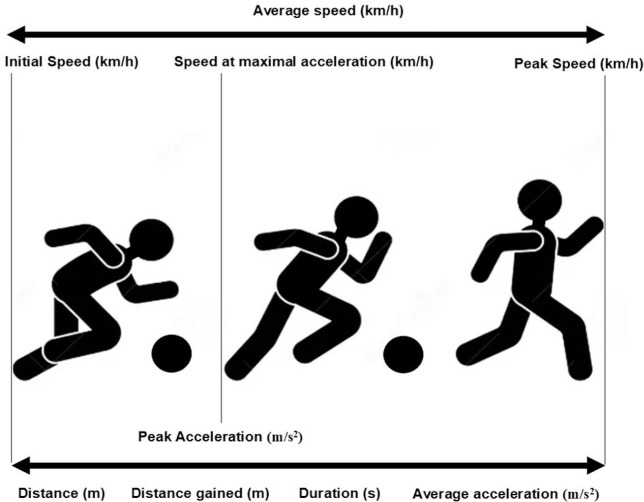
Graphical illustration of the variables of the study.

Firstly, the number of accelerations per minute was calculated considering the total number of accelerations and the total number of minutes of exposure in each match. Secondly, the locomotor characteristics of each acceleration were described by nine variables (Figure 2) that evaluate distance (in metres), duration (in seconds), intensity (in m · s^−2^) and speed (in km · h^−1^):

– Distance (metres): total distance covered during the acceleration.
– Distance gained (metres): total distance covered from the initiation point of the acceleration to its termination, considering the direction relative to the opposing goal line. This distance is classified as positive when the acceleration occurs in the direction of the opposing goal line, indicating forward movement. Conversely, it is considered negative if the acceleration occurs toward the player’s own goal line, reflecting backward or retreating movement.
– Duration (seconds): Total time that the acceleration lasted.
– Peak acceleration (m · s^−2^): Highest rate of change in velocity a player achieved during the acceleration process of each action.
– Average acceleration (m · s^−2^): Mean rate of change in velocity a player achieved during the acceleration event.
– Initial speed (km · h^−1^): Player’s velocity at the moment he began accelerating.
– Peak speed (km · h^−1^): Highest velocity a player reached during the acceleration phase of each event.
– Speed at peak acceleration (km · h^−1^): Speed at the exact moment when the player is exerting the highest rate of acceleration.
– Acceleration average speed (km · h^−1^): Mean velocity during the entire acceleration phase, calculated from the start to the end of the event.

In addition to acceleration variables, contextual variables such match location (home vs away) and ranking of the opponent at the end of the season (in quartiles: first: 1^st^ to 5^th^ position; second: 6^th^ to 10^th^ position; third: 11^th^ to 15^th^ position; fourth: 16^th^ to 20^th^ position) were included in the analysis based on previous studies [9, 37]. By incorporating these variables, the analysis ensures that acceleration metrics are interpreted within the broader competitive context, leading to more accurate and meaningful insights.

### Statistical analysis

The data of each acceleration were transferred from Mediacoach to a .csv database which was organised in Microsoft Excel. All statistical analyses were carried out using the software IBM SPSS Statistics Version 28.0. Firstly, descriptive values of accelerations per minute were calculated. The normality and homogeneity of the variances were confirmed using the Kolmogorov-Smirnov and Levene statistical tests. Therefore, one-way analysis of variance (ANOVA) analysis and the Bonferroni post-hoc test were performed to analyse possible differences between different playing positions regarding the number of intense accelerations per minute.

Secondly, a generalized mixed linear model was carried out to explore the effects of the playing position (fixed effects) on the locomotor characteristics of accelerations, considering the player ID as a random effect. Thus, the “player ID effect” represented the specific player characteristics that may influence the acceleration performance and account for the non-independence of the data [38]. Additionally, contextual variables, such as match location and the opponent’s ranking at the end of the season, were included in the model as fixed effects.

Graphic charts depicting the estimated means (EM) and 95% confidence intervals (95% CI) were used to illustrate players’ acceleration profiles by playing position. Pairwise comparisons of the estimated means were conducted using Fisher’s least significant difference test, with the significance level set at p < 0.05.

## RESULTS

Table 1 shows the number of accelerations per minute per player position and the differences among playing positions. Overall, players performed 1.88 ± 0.36 relative accelerations per minute (> 50% of maximal acceleration-speed profile) and a significant effect of playing position was found. Specifically, CD exhibited fewer accelerations per minute (1.59 ± 0.25) than the rest of the playing positions (CD vs CM: p = 0.006; CD vs AM: p = 0.004; CD vs W: p = 0.039). CM and AM were the playing positions with higher number of accelerations per minute, although no differences were found in comparison with W and FW.

**TABLE 1:** Descriptive analysis of the number of accelerations according to the playing position.

Playing position	Mean (acc/min)	SD	Minimum	Maximum
**CD**	1.59_CM, AM, W,_	0.25	1.04	2.05
**FB**	1.81	0.41	2.03	2.91
**CM**	2.09_CD_	0.28	1.67	2.71
**AM**	2.06_CD_	0.34	1.40	2.46
**W**	1.97_CD_	0.34	1.34	2.57
**FW**	1.81	0.35	1.40	2.26
**All**	1.88	0.36	1.04	2.91

SD = standard deviation; _CD_ = different from CD; _FB_ = different from FB; _CM_ = different from CM; _AM_ = different from AM; _W_ = different from W; _FW_ = different from FW. CD vs CM: p = 0.006; CD vs AM: p = 0.004; CD vs W: p = 0.039).

Tables 2 and 3 present the standardised regression coefficients for each model. A significant effect of playing position was observed across all acceleration characteristics and acceleration speeds. Specifically, W, FB, FW and AM showed higher coefficients than CDs for both acceleration distance (Coeff = 8.13; SE = 0.08) and duration (Coeff = 2.08; SE = 0.01). In terms of distance gained with each acceleration, an increasing trend was observed as the playing positions become more offensive oriented, with FB, CM, AM, W and FW showing progressively higher coefficients than CD. In addition, CM and AM exhibited lower coefficients than CD in relation to peak acceleration (Coeff = 2.94; SE = 0.03) and average acceleration (Coeff = 1.69; SE = 0.02), while FW presented a significantly higher coefficient in both variables than the rest of the playing positions.

**TABLE 2:** Standardised regression coefficients for acceleration distance, distance gained, duration and acceleration intensity according to playing positions, considering the effect of contextual variables (opponent ranking, match location and player ID).

	Distance (m)	Distance gained (m)	Duration (s)	Peak acceleration (m · s^−2^)	Average acceleration (m · s^−2^)

	Coeff (SE)	Coeff (SE)	Coeff (SE)	Coeff (SE)	Coeff (SE)
**Intercept**	8.13 (0.08)***	-1.98 (0.15)***	2.08 (0.01)***	2.94 (0.03)***	1.69 (0.02)

**Playing position**					
**CD**					
**FB**	0.62 (0.08)***	0.89 (0.15)***	0.08 (0.01)***	-0.02 (0.02)	-0.03 (0.02)
**CM**	0.04 (0.09)	0.99 (0.17)***	0.01 (0.01)	-0.15 (0.03)***	-0.08 (0.02)***
**AM**	0.46 (0.13)***	2.77 (0.28)***	0.06 (0.02)**	-0.17 (0.06)**	-0.10 (0.03)**
**W**	0.99 (0.12)***	3.92 (0.25)***	0.11 (0.02)***	0.07 (0.05)	0.00 (0.02)
**FW**	0.56 (0.14)***	4.72 (0.27)***	0.07 (0.02)**	0.17 (0.06)**	0.06 (0.03)*

**Opponent ranking**					
**1^st^ to 5^th^**					
**6^th^ to 10^th^**	0.03 (0.28)	0.24 (0.04)***	0.01 (0.01)	0.01 (0.01)	-0.00 (0.00)
**11^nd^ to 15^th^**	-0.15 (0.02)***	0.22 (0.04)***	-0.02 (0.01)***	0.05 (0.01)***	0.03 (0.00)***
**16^th^ to 20^th^**	-0.02 (0.03)	0.49 (0.04)***	-0.01 (0.01)	0.04 (0.01)***	0.02 (0.00)***

**Match location**					
**Away**					
**Home**	-0.01 (0.01)	0.23 (0.03)***	-0.01 (0.00)	0.00 (0.01)	0.01 (0.00)

**Random effects**					
**Player ID**	0.10*	0.42 **	0.002*	0.022**	0.006**

*** = p < 0.001;

** = p < 0.01;

* = p < 0.05

**TABLE 3:** Standardised regression coefficients for acceleration initial speed, peak speed, speed at maximal acceleration and average according to playing positions and considering the effect of contextual variables (opponent ranking, match location and player ID).

	Initial speed (km · h^−1^)	Peak speed (km · h^−1^)	Speed at peak acceleration (km · hM^−1^)	Average speed (km · h^−1^)

	Coeff (SE)	Coeff (SE)	Coeff (SE)	Coeff (SE)
**Intercept**	6.65 (0.75)***	18.25 (0.10)***	12.08 (0.06)***	14.02 (0.09)***

**Playing position**					
**CD**					
**FB**	0.37 (0.08)***	0.47 (0.08)***	0.40 (0.07)***	0.46 (0.07)***
**CM**	0.73 (0.09)***	-0.12 (0.09)	0.24 (0.08)**	0.15 (0.08)
**AM**	0.88 (0.12)***	0.38 (0.20)	0.56 (0.11)***	0.52 (0.12)***
**W**	0.62 (0.11)***	1.25 (0.17)***	0.75 (0.10)***	0.97 (0.11)***
**FW**	0.26 (0.12)*	0.95 (0.18)***	0.42 (0.11)***	0.54 (0.12)***

**Opponent ranking**					
**1^st^ to 5^th^**					
**6^th^ to 10^th^**	-0.01 (0.02)	-0.01 (0.02)	-0.02 (0.02)	0.20 (0.02)
**11^nd^ to 15^th^**	-0.14 (0.03)***	-0.03 (0.02)	-0.07 (0.02)***	-0.08 (0.02)***
**16^th^ to 20^th^**	-0.10 (0.03)***	0.01 (0.02)	-0.05 (0.02)*	-0.03 (0.02)

**Match location**					
**Away**					
**Home**	0.03 (0.01)	0.04 (0.01)*	0.03 (0.02)	0.03 (0.02)

**Random effects**					
**Player ID**	0.081*	0.223**	0.053**	0.089**

*** = p < 0.001

** = p < 0.01

* = p < 0.05

Additionally, a significant effect of playing position was identified for all variables concerning acceleration speeds (Table 3). FB, CM, AM, W and FW showed higher coefficients than CD in relation to initial speed (Coeff = 6.65; SE = 0.75). Regarding peak speed obtained during the acceleration (Coeff = 18.25; SE = 0.010), FB, W and FW exhibited higher coefficients than CD, while no significant differences were found between CM, AM and CD. Furthermore, FB, CM, AM, W and FW showed higher coefficients than CD in relation to speed at peak acceleration (Coeff = 12.08; SE = 0.06). Finally, FB, AM, W and FW showed higher coefficients than CD regarding average speed (Coeff = 14.02; SE = 0.09).

As for contextual variables, the results highlight significant differences in distance gained during matches against top-ranked opponents (1^st^–5^th^) compared to lower-ranked teams (p < 0.001). Specifically, the distance gained was significantly greater when playing against teams ranked 6^th^–10^th^ (+0.24 m), 11^th^–15^th^ (+0.22 m), and 16^th^–20^th^ (+0.49 m). These differences were especially pronounced in matches played at home (+0.23 m), suggesting a potential contextual influence of match location on performance metrics (p < 0.001).

Conversely, initial speed and speed at peak acceleration decreased when facing lower-ranked teams (11^th^–15^th^ [-0.14 km · h^−1^ and -0.07 km · h^−1^, respectively] and 16^th^–20^th^ [-0.10 km · h^−1^ and -0.05 km · h^−1^, respectively]) compared to matches against topranked teams (1^st^–5^th^). Additionally, greater intensity was observed in average accelerations (+0.03 m · s^−2^ and +0.02 m · s^−2^, respectively) and peak accelerations (+0.03 m · s^−2^ and +0.04 m · s^−2^, respectively) when playing against lower-ranked teams (11^th^–15^th^ and 16^th^–20^th^).

The analysis of random effects (Tables 2 and 3) highlights the variability in distance- and speed-related acceleration metrics. Player-specific random effects were statistically significant but accounted for a relatively small proportion of variance. For distance metrics, random effects for distance covered (0.10) and distance gained (0.42) indicated moderate variability due to player differences, while speed metrics such as initial speed (0.081) and peak speed (0.223) showed similar patterns.

**FIG. 3:**
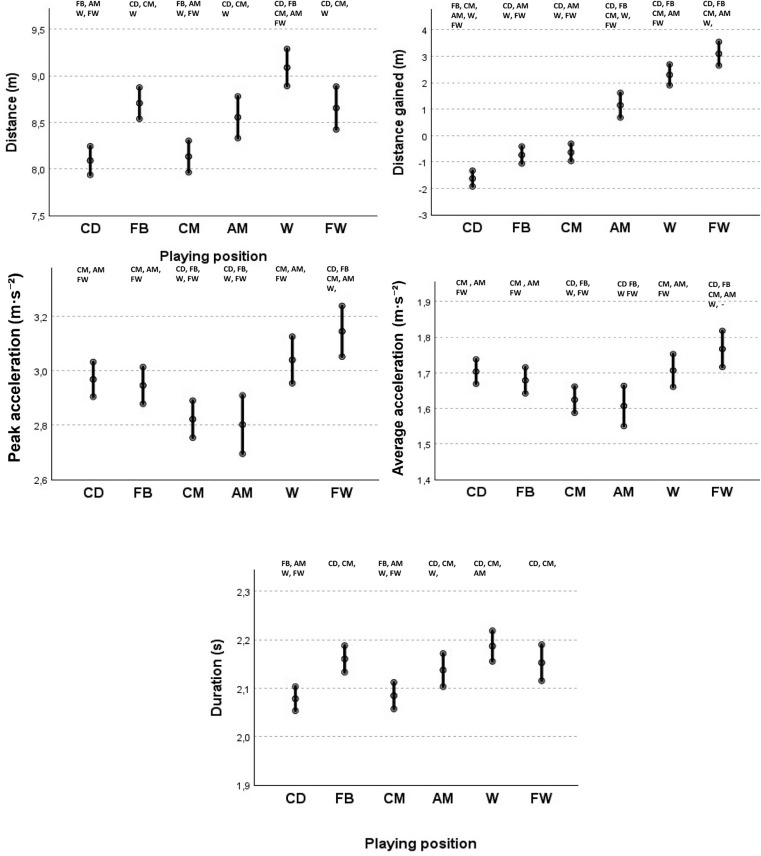
Estimated means and 95% confidence intervals for acceleration distance, distance gained, duration and acceleration intensity according to playing positions, considering the effect of contextual variables (opponent ranking, match location and player ID). CD = different from CD; FB = different from FB; CM = different from CM; AM = different from AM; W = different from W; FW = different from FW.

Figure 3 depicts the EM, and 95% CI of the variables related to acceleration characteristics according to the playing position. W emerged as the position with the greatest distance per acceleration (EM = 9.08 m; 95% CI: 2.95–3.12), significantly exceeding all other positions, followed by FB, FW, AM, CM, with CD registering the lowest distance (EM = 8.09; 95% CI: 7.93–8.24). Regarding the distance gained relative to the opposing goal line, CD, FB, and CM recorded negative values, indicating that a significant proportion of the accelerations performed by these players were directed towards the own goal. In contrast, AM, W, and FW displayed positive distance values, suggesting a forward-oriented acceleration pattern characteristic of these playing positions.

As for acceleration intensity, FW exhibited the highest peak acceleration (EM = 3.14 m · s^−2^; 95% CI: 3.05–3.24) and average acceleration (EM = 1.76 m · s^−2^; 95% CI: 1.71–1.81) surpassing all other positions. W, CD, FB exhibited similar values for both variables, while CM and AM recorded the lowest values in terms of both peak and average acceleration. Finally, W exhibited the longest acceleration durations (EM = 2.18 s; 95% CI: 2.15–2.21), followed by FB, FW and AM, whereas CM and CD registered the shortest durations.

**FIG. 4:**
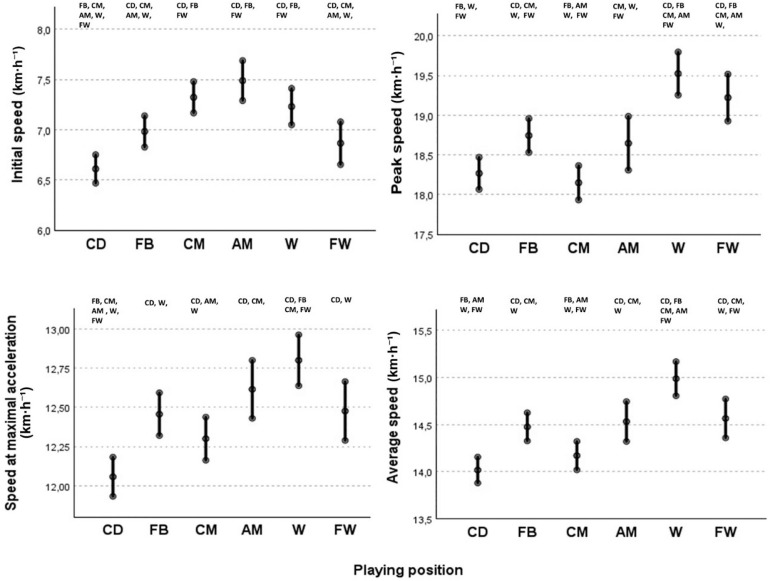
Estimated means and 95% confidence intervals for initial speed, peak speed, speed at maximal acceleration and average speed according to playing positions, considering the effect of contextual variables (opponent ranking, match location and player ID) CD = different from CD; FB = different from FB; CM = different from CM; AM = different from AM; W = different from W; FW = different from FW.

Figure 4 presents the EM and 95% CI for variables related to acceleration speed by playing position. AM (EM = 7.5 km · h^−1^; 95% CI: 7.29–7.68), followed closely by CM and W, initiated the accelerations at significantly higher speeds compared to FB, FW and CD (EM = 6.60 km · h^−1^; 95% CI: 6.46–6.75), which had the lowest initial speeds, in that order. Moreover, W demonstrated the highest peak speed during accelerations (EM = 19.5 km · h^−1^; 95% CI: 19.25–19.80) significantly outperforming other positions, followed closely by FW (EM = 19.2 km · h^−1^; 95% CI: 18.9–19.5), which also exceeded the other playing positions in speed. CD and CM registered the lowest peak speeds per acceleration. Furthermore, W were also the players that exhibited higher speed at peak acceleration (EM = 12.8 km · h^−1^; 95% CI: 12.6–12.9) and average speed (EM = 14.9 km · h^−1^; 95% CI: 14.8–15.2), ahead of FW, AM and FB, while CM and CD had the lowest values for both variables.

## DISCUSSION

This study aimed to evaluate the locomotor characteristics of intense relative accelerations (> 50% of maximal acceleration-speed profile) in terms of distance, magnitude and speed, according to playing positions in top-ranked Spanish football players. The current findings provide new insights into the acceleration profile of top-level footballers, as this study is the first in the literature to include an individualised threshold for categorizing an action as an acceleration (reaching at least > 50% of the acceleration-speed profile) and considering the initial speed at which the acceleration begins.

Generally, players performed approximately two intense accelerations per minute during competition. Moreover, accelerations cover short distances (on average 8–9 metres), last approximately 2 seconds, and show an average peak intensity of 2.8–3.2 m · s^−2^, alongside an average acceleration of 1.6 to 1.8 m · s^−2^. It is noteworthy that accelerations started at an initial speed of 6.5 to 7.5 km · h^−1^, reaching peak speeds of 18–20 km · h^−1^, with a speed at peak acceleration approximating 12 to 13 km · h^−1^.

These findings illustrate how moderate-intensity accelerations are characterized by very short durations and distances, playing a key role in the intermittent nature of football [39]. Furthermore, these accelerations occur more frequently than high-intensity sprints, which have been shown to occur approximately 10–15 times per match [40]. These findings represent valuable information to strength and conditioning coaches, as it may be used to create specific training scenarios to develop the most common type of intense accelerations. In fact, the locomotor characteristics of these actions are different from those performed to improve maximal running speed, which requires greater distances, longer duration and higher running speeds.

Similarly, previous research has reported that most accelerations originate from a starting velocity below the threshold for high-velocity running and do not reach this threshold during the movement [12]. Specifically, the current findings regarding initial speed are slightly higher than those reported by Oliva-Lozano et al. [41], who observed an initial speed of 5 to 6 km · h^−1^ both for high-intensity (> 3 m · s^−2^) and low-intensity accelerations (< 3 m · s^−2^). In this context, the study of De Hoyo et al. [31] analysed players’ relative high-intensity accelerations (> 75% of maximum acceleration [acc_max_]) in a Spanish LaLiga team, revealing that high-intensity accelerations starting above 14 km · h^−1^ were the most common during matches, accounting for more than 40% of all instances. These findings underscore the importance of accounting for initial speed when analysing accelerations in football, as was also highlighted by the study of Oliva-Lozano et al. [41]. Additionally, the reliance on absolute acceleration thresholds may lead to an underestimation of actions when the initial speed is high or an overestimation when the initial speed is low [23]. For this reason, the methodological approach used in this investigation combining the identification of accelerations with respect to the player’s maximal acceleration-speed profile during the season (i.e., only those with > 50% of this value) and the analysis of initial speeds may be an addition to the literature on this topic, as it allows a more accurate analysis of player load during matches.

Additionally, this study confirms that characteristics of the acceleration profile of top ranked players are position- dependent. Firstly, CD (1.59 ± 0.25) exhibited significantly fewer accelerations per minute than players in other positions. In contrast, CM (2.09 ± 0.28) and AM (2.06 ± 0.34) registered the highest acceleration frequency, although no significant differences were found when compared with W (1.97 ± 0.34) and FW (1.81 ± 0.35). Regarding acceleration metrics, W covered the greatest acceleration distance, while W and FW recorded the highest values for distance gained, peak acceleration, and average acceleration. In contrast, CD and CM registered the lowest values for acceleration distance and duration, while CM and AM had the lowest peak and average acceleration, highlighting the distinct acceleration profiles of internal playing positions. In terms of acceleration speed metrics, the present study revealed that AM and CM, followed by W, accelerated from higher initial speeds, whereas FW and CB recorded the lowest values in this metric. Notably, W exhibited the highest values for peak speed, speed at maximal acceleration, and average speed, followed by FW, AM, and FB, forming the next group of positions with elevated acceleration speed. In contrast, CD and CM had the lowest speed metrics during acceleration actions.

These findings emphasize the varying acceleration profiles across different positional roles, which is crucial for understanding the physical demands of football. In this regard, W and FW were the positions with the highest intensity values, with W leading in distance and speed, while FW excelled in acceleration intensity and average acceleration. In contrast, CD and CM recorded the lowest values, with CD displaying the lowest speed performance and CM the lowest acceleration performance. Meanwhile, FB exhibited moderate levels in distance, duration, peak acceleration, average acceleration, and speed-related variables, whereas AM showed moderate values in distance, duration, and speed variables.

The higher acceleration distance and speed of W have been documented in previous research studies [31, 41]. Oliva-Lozano et al. [41] observed that W covered the greatest distance in accelerations within a Spanish professional team, followed by FB and FW. Similarly, De Hoyo et al. [31] revealed that FW, W, and FB performed a greater number of high-intensity accelerations than CD and CM in another Spanish LaLiga team. Dalen et al. [42] also reported that W and FB accelerated more frequently than CD, CM and FW in a Norwegian first division team. This may be explained by the positional demands of playing on the flanks, which often require covering more ground, particularly for positions that involve both offensive and defensive responsibilities. From a practical standpoint, these data suggest that the accelerative capacity should be trained taking into account the playing position. While W, FB, FW and AM would benefit from exercises designed to cover longer distances and to achieve greater running speeds, CD and CM should focus on shorter-distance exercises that replicate accelerations from lower initial speeds, aligning with the typical demands of these specific roles.

As for the spatial orientation, significant differences were found among all playing positions, with CD, FB, and CM registering negative distance, which indicates that these players performed more accelerations oriented towards their own goal line. In contrast, FW, W, and AM exhibited a forward orientation when accelerating, highlighting a more offensive tactical role. These findings underscore the importance of tactical specificity when training accelerations so that some playing positions may require more defending or offensive accelerations. It is relevant to find that FB, despite the position’s offensive importance in Spanish football [43], perform a great number of acceleration actions in a backward direction. This suggests that FB play a dual role, frequently transitioning between defensive and attacking phases [44]. While these players are often involved in offensive plays, the defensive responsibilities require frequent backward accelerations, likely due to tracking opponents, repositioning, or recovering defensive shape. This reinforces the need for position-specific acceleration training to optimise performance in both attacking and defensive scenarios.

Regarding the acceleration initial speed, the present study revealed that AM and CM accelerated from higher speeds, while FW and CB registered the lowest values in this metric. These results highlight the variation in acceleration patterns based on playing position and can be linked to the different running demands imposed on players during matches. Previous studies have emphasised that midfielders cover the greatest distance during matches [45], spending a significant percentage of time at moderate running speeds [46, 47]. This increased movement may require these players to accelerate more and from higher initial speeds, which in turn reduces the acceleration intensity. However, this finding contrasts with some evidence suggesting that players in wide positions, such as FB and W, perform the highest number of accelerations [31, 42]. This discrepancy might be partially explained by the use of absolute acceleration thresholds (e.g., > 2 m · s^−2^) in earlier studies [42], which could underestimate high-intensity accelerations for players who often accelerate from higher initial speeds [31]. Nevertheless, the difference remains even when using relative thresholds based on individual acceleration-speed profiles [31]. Additionally, the variation in specific relative thresholds (e.g., 50% acc_max_ vs 75% acc_max_) may further influence these observations. Lower thresholds, such as 50% acc_max_, may favour the identification of high-intensity events for midfielders, who have been reported to sustain higher metabolic power outputs and longer durations of submaximal efforts during competition [48].

Conversely, CD and FW seem to have more intermittent movement patterns, covering less total distance and spending more time walking or at low speeds than other playing positions [49], which demands greater intensity to transition from low to high speeds. In line with this interpretation, this study found that FW exhibited the highest peak and average accelerations, while CD displayed higher acceleration intensity values than CM and AM. These findings highlight that FW and CD require a high capacity to accelerate from slow speeds, which demands significant locomotor efforts and results in a distinct acceleration profile compared to other playing positions. Specifically, the current study shows that FW in Spanish top-ranked football teams possess exceptional acceleration capacity, which may be crucial for offensive actions aimed at beating defenders and creating goal-scoring opportunities.

Our findings extend this existing knowledge by not only examining total acceleration distance or frequency but also analysing the specific locomotor characteristics of each effort. In this regard, W demonstrated the highest conditional demands related to acceleration, as these players covered the greatest distance per acceleration and achieved the highest peak speed throughout the acceleration phase, including at the moment of peak acceleration for each action. Moreover, W maintained the highest average speed, potentially influenced by the highest peak speed and acceleration [50]. These findings further emphasise the demanding physical profile of players in W positions within top LaLiga football teams. Additionally, the high acceleration performance observed in FW, FB, and AM aligns with the increasing physical demands of modern football, highlighting the evolving athletic requirements of these positions [4, 37].

Finally, contextual factors significantly influenced the acceleration demands. Players tended to show reduced acceleration demands, such as decreasing initial speed, average speed, distance per action and duration, when facing lower ranked opponents. This adjustment may reflect tactical strategies, such as a more offensive-oriented behaviour [51–53]. Additionally, the large increase in distance gained might be attributed to more forward-oriented efforts, not only offensively with more attacking attempts, but also during defensive phases, driven by pressing strategies. In contrast, match location barely influenced the acceleration performance except for distance gained and high peak speed during accelerations, which showed higher values in home matches. These findings highlight a more forward-oriented direction in the physical efforts, probably due to a more attacking intention in home matches, which could also exhibit higher intensity specifically in peak speeds reached during accelerations. These findings align partially with existing literature, which suggests that playing at home tends to increase high-intensity efforts [9, 54, 55], although our investigation did not find significant differences in workloads regarding the rest of the variables related to the acceleration profile of players.

This study has limitations that need to be acknowledged. Firstly, the results should be interpreted with caution, as this study focused on a specific sample of professional Spanish football players from the top four teams in the competition. Consequently, the findings may not be directly applicable to players from lower-ranked teams in the same league, younger athletes, female footballers, or players in other competitions [18]. Secondly, the video-tracking system used in this study is only used in top-tier leagues and thus is not directly transferable to other football contexts. Thirdly, although this study used a new methodological approach to quantify accelerations per playing position, it should be noted that some short and intense locomotor actions such as vertical jumps, rotations and decelerations were not analysed. Nevertheless, the large sample size, the inclusion of contextual variables, as well as the new metrics analysed, provide strong consistency and novel contributions to the existing literature.

Fitness coaches should design specific training programmes considering the locomotor characteristics of accelerations in top football players. These accelerations do not generally reach nearto-maximum speed but have high mechanical and muscle demands. It is also crucial to consider the initial speed when analysing the intensity of accelerations, as well as to understand the different patterns of acceleration according to the playing position. Overall, the data reveal that midfielders are required to accelerate from higher initial speeds, while W exhibited the greatest acceleration challenges, having longer acceleration phases with extended durations, and higher peak speeds. Also, FW were the players with the greatest peak acceleration capacity and total distance gained, likely because the actions of these players are predominantly linear towards the rival goal. On the other hand, CD and FB need to be prepared for frequent accelerations while defending, often sprinting toward their own goal. Finally, practitioners should consider contextual factors when analysing acceleration data, so that playing against higher-ranked opponents increases the acceleration demands, while playing at home slightly increases the peak speed and the distance gained with accelerations. This information is useful to better interpret player workloads and design training and recovery strategies.

## CONCLUSIONS

In conclusion, this study highlights how acceleration demands vary across playing positions in elite Spanish football. Midfielders (AM and CM) tend to accelerate from higher initial speeds, while FW and W generate the most intense accelerations. Also, W cover the greatest distance and duration in acceleration actions, while central players, particularly CM and CD, exhibit the lowest values. These findings emphasise the unique physical demands and movement patterns associated with each position, offering valuable insights for training and performance optimization.
